# A robust design for identification of the Parasite Clearance Estimator

**DOI:** 10.1186/1475-2875-12-410

**Published:** 2013-11-13

**Authors:** Kris M Jamsen, Stephen B Duffull, Joel Tarning, Ric N Price, Julie A Simpson

**Affiliations:** 1 Centre for Molecular, Environmental, Genetic and Analytic Epidemiology, Melbourne School of Population and Global Health, The University of Melbourne, Parkville, Australia; 2 School of Pharmacy, University of Otago, Dunedin, New Zealand; 3 Mahidol-Oxford Tropical Medicine Research Unit, Mahidol University, Bangkok, Thailand; 4 Centre for Tropical Medicine, Nuffield Department of Clinical Medicine, University of Oxford, Oxford, UK; 5 Global Health Division, Menzies School of Health Research and Charles Darwin University, Darwin, Australia; 6 WorldWide Antimalarial Resistance Network (WWARN), Oxford, UK

## Abstract

**Background:**

Anti-malarial efficacy needs to be monitored continually to ensure optimal dosing in the face of emerging anti-malarial drug resistance. The efficacy of artemisinin based combination therapies (ACT) is assessed by repeated measurements of parasite density in the blood of patients following treatment. Parasite density is measured from a capillary or venous blood sample, but this can be logistically and ethically challenging if multiple samples are required within a short time period. The aim of this work was to apply optimal design theory to derive clinically feasible blood sampling schedules from which parasite clearance could be defined using the Parasite Clearance Estimator (PCE), a recently developed tool to identify and quantify artemisinin resistance.

**Methods:**

Robust T-optimal design methodology was applied to offer a sampling schedule that allows for discrimination across models that best describe an individual patient’s parasite-time profile. The design was based on typical parasite-time profiles derived from the literature combined with key sampling constraints of no more than six samples per patient within 48 hours of initial treatment. The design was evaluated with a simulation-estimation procedure that implemented the PCE.

**Results:**

The optimal sampling times (sampling windows) were: 0 (0 to 1.1), 5.8 (4.0 to 6.0), 9.9 (8.4 to 11.5), 24.8 (24.0 to 24.9), 36.3 (34.8 to 37.2) and 48 (47.3, 48.0) hours post initial treatment. The simulation-estimation procedure showed that the design supported identification of the appropriate method by the PCE to determine an individual’s parasite clearance rate constant (the main output calculation from the PCE).

**Conclusions:**

The proposed sampling design requires six samples per patient within the first 48 hours. The derived design requires validation in a real world setting, but should be considered for future studies that intend to employ the PCE.

## Introduction

The artemisinin derivatives remain potent agents in the anti-malarial armamentarium. However, their efficacy is under threat from emerging evidence documenting reduced parasite sensitivity in Cambodia [1,2]. This alarming finding provides motivation for conducting more efficacy and pharmacokinetic-pharmacodynamic (PK-PD) studies of these important drugs to monitor their efficacy and re-assess current dosing regimens.

A key pharmacodynamic measure of anti-malarial drug efficacy is the measurement of parasite density in the peripheral blood, usually determined by a finger prick sample and examination of a drop of blood by microscopy. A variety of parameters are available for quantifying the drug efficacy on parasite dynamics. Repeated measures of parasitaemia can be used to determine the parasite clearance time, defined as the time from the start of treatment to a parasite count below the microscopy limit of detection. Analytical approaches to quantifying an individual’s parasite clearance time vary although recently interest has focused on the Parasite Clearance Estimator (PCE) [3], a tool developed specifically to calculate a rate constant (the parasite clearance rate constant, PCRC) from repeated measures of parasitaemia. In brief, the PCE calculates the PCRC by first fitting linear, quadratic and cubic regression models to an individual’s observed (log) parasite-time data, and identifies which of these models provides the best fit. If the best model is linear, the PCE declares the PCRC as the absolute value of the estimated slope. If the best model is quadratic or cubic, the PCE performs an algorithm to determine if the model’s predictions exhibit an initial delay in parasite decline, and the PCRC is calculated as the absolute value of the estimated slope from a linear model fitted to the subset of best model predictions that display linearity over time. The PCRC is considered a robust proxy measure for an individual’s parasite clearance time [4].

For the PCE to determine the appropriate method for calculating an individual’s PCRC, blood sampling needs to be frequent enough to provide means for model discrimination (across log-linear, quadratic and cubic parasite-time profiles) and allow the PCE to detect delays in parasite reduction. Determining blood sampling schedules in patients with uncomplicated falciparum malaria treated with artemisinin-based combination therapies (ACT) can be challenging, as it can be logistically and ethically difficult to impose intensive schedules over the timespan where parasites are above the limit of microscopic detection (approximately 48 hours post initial treatment [5-8]). Therefore a sampling schedule for the PCE must be clinically feasible within the first 48 hours of treatment and offer sufficient information for appropriate implementation.

T-optimal designs offer a sampling schedule that allows for discrimination across competing models. In brief, an iterative procedure is used to achieve sampling times that capture the largest differences across the specified competing profiles. To date, analytical approaches to designing efficacy (i.e. pharmacodynamics, or PD) studies, such as optimal design methods, have not been applied to the study of treatment response following anti-malarial therapy. The aim of the current study was to use optimal design methodology to determine a clinically feasible sampling design for future studies that intend to use the PCE.

## Methods

### Determination of the design for the Parasite Clearance Estimator

**Figure 1:**
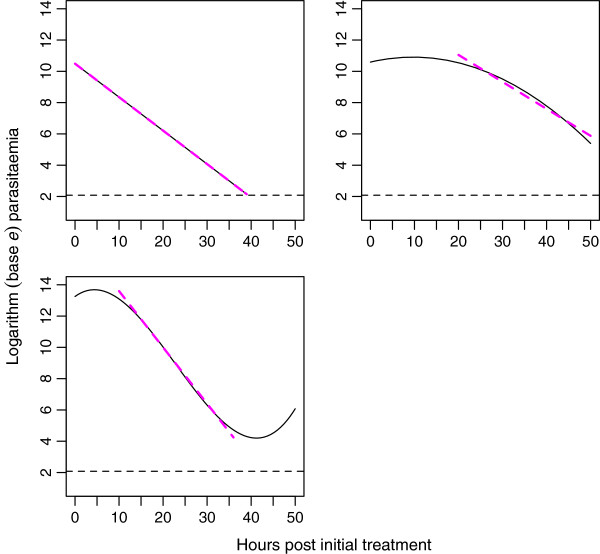
**Graphical representation of the Parasite Clearance Estimator.** The black lines represent typical log parasitaemia-time profiles (linear, top left; quadratic, top right; cubic, bottom left), the dashed horizontal line indicates the microscopic limit of parasite detection and the parasite clearance rate constant is the absolute value of the slope of the dashed maroon line.

The PCE is defined on the basis of the PCRC, which is the slope of the log (base *e*) parasitaemia-time relationship for an individual patient, accounting for any initial delay in parasite reduction [3]. Figure 1 provides a visual aid to the PCE. For full details of the PCE, including the algorithm it performs to detect an initial delay in parasite reduction, see Additional file 1 and [3].

As a key aspect of the PCE is determining a “best” model that describes the log parasite-time relationship for an individual, a robust T-optimal design was determined for future studies that intend to use the PCE. A robust T-optimal design determines a sampling schedule that provides means for model discrimination and has the additional benefit of not assuming one of the models is “true”. For further technical details of robust T-optimality see [9]. The design was derived based on the following specifications: (i) six samples per person over the first 48 hours of treatment (the suggested minimum for model exploration [3]), and (ii) “typical” quadratic and cubic log parasite-time profiles that were presented graphically in [3] (i.e. Figure one in [3]). Flegg *et al.* also presented a “typical” linear parasite-time profile, but this was not considered in the optimization procedure since sampling times at 0 and 48 hours (both taken routinely) would provide enough information for detection of this model. It was not stated in [3] how any of these “typical” profiles were derived, but it was assumed that they were accurate summaries of observed data. Since parameter values for the typical profiles were not reported in [3], parameter values for the quadratic and cubic profiles were obtained by digitizing points from their respective plots in [3] and fitting quadratic and cubic regression models (respectively) to these digitized data in Stata [10]. The results from these analyses are given below in Equation 1. In the equation, *P* represents total parasite count and *t* indicates time (hours post initial treatment).

(1) $$\begin{array}{lll} \log(P)=10.6 & +0.066t-0.0034t^{2}\; & \;\\ & +\varepsilon ,\;\;\;\varepsilon ∼N(0,0.40^{2})\; & \;\\ \log(P)=13.3 & +0.207t-0.026t^{2}+0.00038t^{3}\; & \;\\ & +\varepsilon ,\;\;\;\varepsilon ∼N(0,0.40^{2})\; \end{array}$$

Using these results, the following algorithm developed by Vajjah and Duffull [9] was written and implemented in R [11]: 

1. Log parasite counts were simulated from the quadratic model (with residual error) at a pre-specified design (0, 6, 10, 24, 36 and 48 h; based on visual inspection of the typical profiles).

2. A cubic model was fitted to the counts simulated from the quadratic model.

3. Simulated annealing was used to find the design points that maximized the residual sum of squared differences between the counts arising from the quadratic and cubic models.

4. Log parasite counts were simulated from the cubic model (with residual error) at the optimized design points obtained from step 3.

5. A quadratic model was fitted to the counts simulated from the cubic model.

6. Simulated annealing was used to find the design points that maximized the residual sum of squared differences between the counts arising from the quadratic and cubic models.

7. Step 1 was repeated, using the design points obtained in step 6 and parameters obtained in steps 5 and 2.

The process was repeated until a convergence of design points in step 6 was achieved between successive iterations. Convergence was declared when the maximum difference for any design point was less than 0.1 hours.

To provide flexibility with taking samples in the field, sampling windows, which are time intervals that include the optimal sampling times, were derived. The windows were determined using the evaluation procedure described in the next section.

### Evaluation of the design for the Parasite Clearance Estimator

The derived T-optimal design for the PCE was evaluated using a simulation-estimation procedure to assess the ability of the design to support the PCE in identifying the correct method to calculate an individual’s PCRC. At the time this work was completed the PCE was not publicly available, hence the following procedure was coded and implemented in R: 

1. Log parasite count data were simulated at the optimal sampling times from either a linear, quadratic or cubic model with residual error (based on the analysis of digitized data described above) and between-subject variances (BSVs) on the model parameters. The incorporation of BSVs was done to provide a post-hoc assessment of the robustness of the design, as BSVs were not considered in the optimization procedure. Values for the BSVs were chosen based on plausible simulated log parasite-time profiles (displayed in the Results section; see Additional file 1 for full details of the simulation models). The structural model (linear, quadratic or cubic) the data were simulated from was considered the “true” model.

2. Linear, quadratic and cubic models were fitted to the simulated data in step 1.

3. The model with the lowest AIC in step 2 was considered the “best” model.

4. The procedure to identify the method to calculate the PCRC (steps 2 and 3 from the PCE calculation algorithm in Additional file 1) was adopted.

An individual iteration of this procedure was considered successful if the appropriate method for calculating the PCRC was identified. For example, if the linear model was the “true” model, then the PCE should determine that the PCRC should be the absolute value of the estimated slope from a linear model (with a lag of 0). If the “true” model was quadratic or cubic, then the PCE should detect the lags in these models, and hence determine that the PCRC should be the absolute value of the estimated slope from a linear model fitted to the subset of model predictions that display linearity over time. See Figure 1 for a visual guide. For each “true” model, the simulation-estimation process above was repeated 1000 times (i.e. 1000 individual log parasite-time profiles were simulated and then evaluated) and the percentage of successful runs (i.e. the percentage of the 1000 individuals where the appropriate method for calculating PCRC was identified) was recorded. An acceptable success percentage was set to 85%.

Sampling windows for this design were determined using the evaluation procedure. First, lower and upper bounds for each optimal sampling time were specified based on visual inspection of the typical profiles and clinical feasibility. Second, a random sample was taken from within each window, creating a new design, and this new design was then evaluated with the simulation-estimation procedure described above. This process was repeated 100 times (i.e. 100 designs were evaluated with the simulation-estimation procedure above), and the windows were deemed acceptable if the median percentage of successful runs across the 100 designs was at least 80%.

## Results

**Figure 2:**
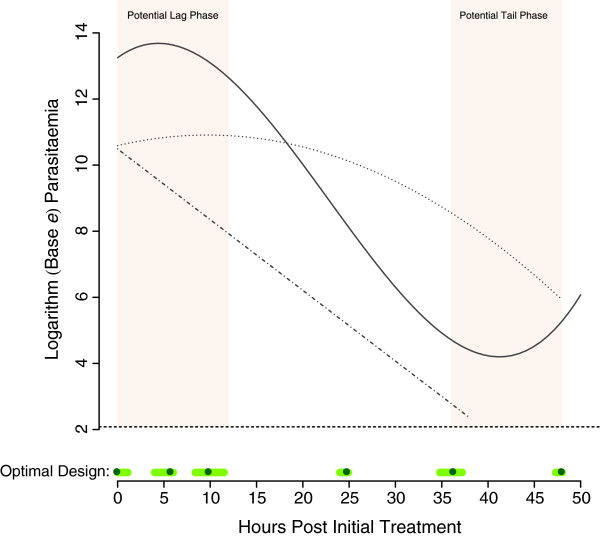
**The derived robust T-optimal design for the Parasite Clearance Estimator.** The solid, dotted and dash-dotted lines represent typical log cubic, quadratic and linear parasite-time profiles (respectively), the dashed horizontal line indicates the microscopic limit of parasite detection and the green circles and light green line segments on the time axis represent the derived optimal sampling times and windows (respectively).

Table 1 displays the optimal sampling times and corresponding sampling windows determined by robust T-optimal methods. Figure 2 displays the design graphically, as well as the competing quadratic and cubic log parasite-time profiles the design was based on (a typical linear log parasite-time profile is also displayed). The optimization procedure captured the potential “lag” and “tail” phases of the profiles as suggested by [3], as well as the routine sampling times of 0, 24 and 48 hours.

**Table 1:** Robust T-optimal design for the Parasite Clearance Estimator

Optimal sampling times*					
**(sampling windows)**					
0.0	5.8	9.9	24.8	36.3	48.0
(0.0, 1.1)	(4.0, 6.0)	(8.4, 11.5)	(24.0, 24.9)	(34.8, 37.2)	(47.3, 48.0)

*Hours post initial treatment.

**Figure 3:**
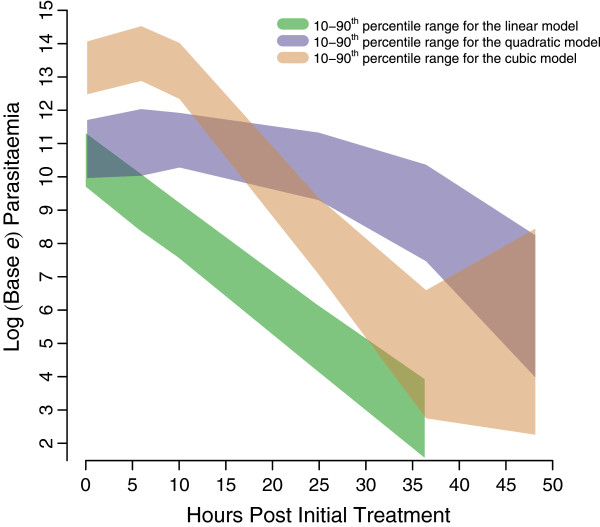
**Summaries of simulated log parasite-time profiles used for the evaluation procedure.** The green, purple and brown shaded regions represent the 10–90 ^th^ percentile ranges of the simulated linear, quadratic and cubic log parasite-time profiles, respectively.

Figure 3 displays summaries of the simulated log parasite-time profiles used for the evaluation procedure and Table 2 reports the results from implementing the procedure. When the “true” log parasite-time profile was specified as a linear model, the appropriate method to calculate the PCRC (the absolute value of the estimated slope from a linear model) was identified in 94% of the simulated individual log parasite-time profiles. When the true log parasite-time profile was specified as quadratic, the appropriate method to calculate the PCRC (the absolute value of the estimated slope from a linear model fitted to the quadratic model predictions after the lag phase) was identified in 96% of the simulated profiles. When the “true” log parasite-time profile was specified as cubic, the correct method for calculating the PCRC (the absolute value of the estimated slope from a linear model fitted to the cubic model predictions between the initial and late lag phases) was identified in 86% of the simulated profiles.

**Table 2:** Evaluation of the robust T-optimal design for theParasite Clearance Estimator

	**Appropriate method to**
**True (simulated) log**	**calculate the parasite clearance**
**parasite-time profile**	**rate constant identified(%) **^**‡**^
Linear	94
Quadratic	96
Cubic	86

^‡^Out of 1000 simulated profiles.

## Discussion

For the first time, optimal design methods have been applied to the study of treatment response following anti-malarial therapy. The derived robust T-optimal design for the PCE provided sufficient means for identifying appropriate sampling from which to calculate the PCRC. The design is clinically feasible, requiring only six samples per patient over the first 48 hours of treatment; two samples less than that suggested previously to detect an initial lag in parasite reduction [4]. However the precise timing of the proposed schedule times may provide logistical challenges in the setting of clinical field trials. Hence further evaluation will be required to demonstrate that the proposed design is robust, efficient and flexible.

The design was based on models that were fitted to digitized data derived from the typical log parasite-time profiles presented in [3]. Although this could be viewed as a limitation (i.e. the models were not fitted to observed data), this was one of the few ways parameter values for the profiles could be obtained, since estimates of the model parameters for these profiles were not reported in [3]. After the completion of this work, a personal communication with Flegg *et al*. revealed that the parameter estimates from the analyses of digitized data were in fact very similar to the parameter estimates from models fitted to observed parasite-time data, thus providing support for the models used for the designs. Another limitation of this study was that the optimization procedure was sensitive to initial design specification. However, the evaluation procedure, which simulated parasite-time profiles from models that incorporated both between- and within-individual variability, showed that the derived design provided sufficient support to the PCE in identifying the appropriate method for calculating an individual’s PCRC. This empirical finding provides support for the design proposed in this paper, and highlights the importance of empirical evaluation in optimal design development. Furthermore, the evaluation procedure enabled derivation and assessment of sampling windows, which may provide more informative windows than those defined by less rigourous methods (e.g. visual inspection alone). Lastly, the derived design is very similar to a reduced sampling scheme determined by Flegg *et al*. that yielded accurate and reliable estimation of parasite half-life via the PCE [12].

## Conclusions

The proposed robust T-optimal design provides guidance for investigators wishing to employ the PCE, now available online from WWARN [13]. As more parasite-time profile data become available from different malaria endemic regions where efficacy of the artemisinin derivatives is declining, the proposed sampling strategy can be re-evaluated and possibly revised to accommodate potentially longer lag phases and/or delayed parasite clearance times.

## Competing interests

This article was submitted to *Malaria Journal* in partnership with Flegg *et al*., who jointly submitted a separate article addressing the topic of this paper using a different methodological approach [12]. The work presented in this paper was completed independently of Flegg *et al*., except where noted in the Discussion. The joint submission of this article and the article by Flegg *et al*. to *Malaria Journal* was agreed upon by all authors of this paper and Flegg *et al*. The authors of this paper declare no other competing interests.

## Authors’ contributions

KMJ and JAS conceived the project. KMJ implemented and evaluated the designs. KMJ wrote the first draft of the manuscript. JAS, SBD, JT and RNP revised the manuscript critically for important intellectual content. All authors read and approved the final manuscript.

## Supplementary Material

Additional file 1 This file provides a description of the Parasite Clearance Estimator and the details of the models used for simulation in the evaluation procedure. Click here for file
