# Supplementary material for: A robust design for identification of the Parasite Clearance Estimator
Source: Malar J. 2013 Nov 13;12:410. doi: 10.1186/1475-2875-12-410 (PMC3831256; doi:10.1186/1475-2875-12-410)
Supplement: Additional file 1 — This file provides a description of the Parasite Clearance Estimator and the details of the models used for simulation in the evaluation procedure. [file 1475-2875-12-410-S1.pdf]

## The Parasite Clearance Estimator

The parasite clearance estimator (PCE) determines the parasite clearance rate constant as follows (the descritption below was modifed from [3]):

1. Different regression models (typically linear, quadratic or cubic) are fit to an individual's log parasite-time data (see Figure 1 for typical log parasite-time profiles), and the "best" model is determined by the Akaike Information Criteria (AIC) or residual sum of squares.

2. If the best model is a linear model, then the parasite clearance rate constant $K$ is simply the estimated slope from this model:

$$E(\log(P)) = \beta_0 + \beta_1 t,$$

   where $P$ is the total parasite count, $t$ is time (hours post initial treatment) and $K = \beta_1$.

3. If the best model is not the linear model (e.g. a quadratic or cubic model resulted in a lower AIC), then:

   (a) for each log parasitaemia predicted by the best model ($E(\log(P_j))$, with $j$ being the $j^{\text{th}}$ measurement of parasite burden at time $t$), calculate the slope $S_j$ between this point and the preceding predicted value:

$$S_j = \frac{E(\log(P_j)) - E(\log(P_{j-1}))}{t_j - t_{j-1}}$$

   For example, if a patient had 6 measurements of parasite burden (including the intial value), there would be five slope values ($S_2, S_3, \ldots, S_6$).

   (b) Determine the $S_j$ with the most extreme negative value, $S_{max}$.

   (c) For each slope $S_j$, calculate $Sn_j = S_j/S_{max}$. Note that a negative $Sn_j$ implies a rise in the parasite count.

   (d) Determine $K$ using the flow chart in Figure S1.

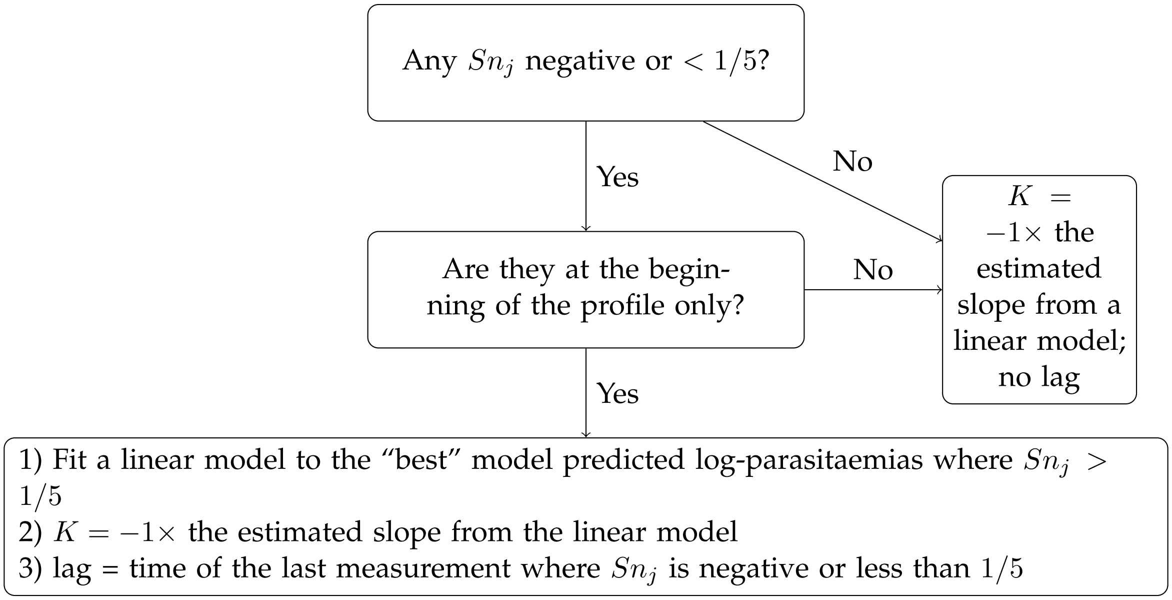


Figure S1: Flow chart for calculation of the parasite clearance rate constant via the Parasite Clearance Estimator

## Simulation models used in the evaluation procedure

The equations below display the simulation models used in the evaluation procedure. For all equations, $P_{ij}$ represents the total parasite count for the $j^{\text{th}}$ observation of the $i^{\text{th}}$ individual, and $t$ indicates time (hours post initial treatment).

$$\begin{aligned}
\log(P_{ij}) &= (10.5 + b_{0i}) - (0.214 + b_{1i})t + \varepsilon_{ij} \\
b_{0i} &\sim N(0, 0.50^2) \\
b_{1i} &\sim N(0, 0.02^2) \\
\varepsilon_{ij} &\sim N(0, 0.41^2)
\end{aligned}$$

$$\begin{aligned}
\log(P_{ij}) &= (10.6 + b_{0i}) + (0.066 + b_{1i})t - (0.0034 + b_{2i})t^2 + \varepsilon_{ij} \\
b_{0i} &\sim N(0, 0.50^2) \\
b_{1i} &\sim N(0, 0.02^2) \\
b_{2i} &\sim N(0, 0.0005^2) \\
\varepsilon_{ij} &\sim N(0, 0.41^2)
\end{aligned}$$

$$\begin{aligned}
\log(P_{ij}) &= (13.3 + b_{0i}) + (0.207 + b_{1i})t - (0.026 + b_{2i})t^2 + 0.00038t^3 + \varepsilon_{ij} \\
b_{0i} &\sim N(0, 0.50^2) \\
b_{1i} &\sim N(0, 0.01^2) \\
b_{2i} &\sim N(0, 0.001^2) \\
\varepsilon_{ij} &\sim N(0, 0.41^2)
\end{aligned}$$
